# Strategic Timing of Resistance Training to Guard Against Antipsychotic-Induced Metabolic Syndrome (START GAAIMS): A Feasibility Study Protocol

**DOI:** 10.12688/hrbopenres.14320.1

**Published:** 2026-04-30

**Authors:** Brian O'Mahony, Kate Pumpa, Donal O'Shea, Karen O'Connor, Michael Norton, Brian O'Donoghue

**Affiliations:** 1 University College Dublin College of Health Sciences, Dublin, Leinster, Ireland; 2 University College Cork, Cork, County Cork, Ireland

**Keywords:** First-episode psychosis, resistance training, metabolic syndrome, antipsychotics, feasibility trial.

## Abstract

**Background:**

Individuals with psychotic disorders face a 20-year mortality gap compared to the general population, largely driven by cardiovascular disease and metabolic syndrome. Antipsychotic medications, while essential, exacerbate this risk through rapid weight gain and metabolic dysregulation, particularly in the first three months of treatment. Resistance training improves body composition and metabolic health, particularly glucose regulation. Its effects on muscle strength and hypertrophy are enhanced by a caloric surplus that, in its absence, would otherwise lead to accumulation of adipose tissue. Its feasibility and efficacy in drug-naïve or minimally treated First-Episode Psychosis (FEP) patients has not been previously explored.

**Methods:**

The START GAAIMS study is a multicentre, two-arm, crossover, randomised controlled feasibility trial. Forty adults (18–65 years) with FEP and < 4 weeks of antipsychotic exposure will be recruited from services in Dublin and Cork. Participants will be randomised (1:1) to an Intervention Group (12-week supervised RT programme + Treatment as Usual [TAU]) or a Control Group (TAU only). After 12 weeks, the control group will crossover to receive the intervention.

**Outcome Measures:**

The primary outcome is the feasibility of the trial design (recruitment, retention, adherence) and a preliminary estimate of the effect size for change in body fat percentage at 12 weeks. Secondary outcomes include muscular power, metabolic biomarkers (glucose, lipids, inflammatory markers), and psychiatric symptoms (BPRS).

**Discussion:**

This study will determine preliminary measures of the effects of resistance training on body composition in people with FEP starting antipsychotic medication, as well as its feasibility and acceptability in this cohort. Findings will inform the design of a definitive RCT to establish RT as a standard co-treatment for metabolic health in early psychosis.

## Introduction

Individuals with psychotic disorders face a significant mortality gap, with a life expectancy reduced by up to 20 years compared to the general population. ^1, 2^ This is largely driven by cardiovascular disease, metabolic syndrome, and obesity. Antipsychotic medications, while essential for managing psychosis, cause significant weight gain and metabolic dysregulation through complex actions on neurochemical pathways that increase appetite and impair glucose metabolism. ^3^

The period immediately following the initiation of antipsychotic treatment is a critical window, as it is associated with the most rapid weight gain. Most of the weight gain that an individual will experience following initiation of antipsychotics will occur in the first three months of treatment. ^4, 5^

Resistance training is a well-established modality for improving body composition, increasing skeletal muscle mass, and enhancing metabolic health, particularly glucose regulation via the GLUT4 transporter system, ^6^ a pathway negatively impacted by antipsychotic medications. ^7^ By channelling the increased caloric intake (driven by antipsychotic-induced orexigenic effects) towards muscle protein synthesis rather than fat storage, resistance training presents a unique therapeutic opportunity. It has the potential to turn a significant adverse effect into a physiological advantage, promoting gains in lean body mass and strength. Resistance training has also been shown to be a feasible intervention in people with psychotic disorders. ^8^

While previous studies have explored exercise in patients with psychotic disorder, they have often used BMI as a primary outcome, which fails to distinguish between fat and muscle mass. Additionally, the vast majority of interventions to prevent antipsychotic-induced weight gain studied to date have included participants with chronic illness who may have had years of antipsychotic exposure. ^9^ No study to date has specifically investigated the potential for resistance training to promote lean body mass gain in a drug-naïve or minimally treated FEP cohort during the critical early phase of treatment.

### Objectives

Primary Objective

To assess the feasibility of the trial design and to obtain a preliminary estimate of the effect size of a 12-week resistance training intervention on **body composition** in people with FEP.

Secondary Objectives

To evaluate the feasibility of recruitment, randomisation, retention, and assessment procedures.

To assess the acceptability and safety of the resistance training intervention and all study procedures.

To gather preliminary data on the effects of the intervention on:

• Physical health outcomes (body composition, muscular power, cardiovascular markers).
• Mental health outcomes (psychotic, depressive, and anxiety symptoms).
• Functioning and quality of life.
• Metabolic and inflammatory biomarkers.
• To explore the effects on weight gain of resistance training, when this intervention is delayed.
• To explore the lasting impact on weight gain trajectory of a resistance training intervention.

The trial will also allow for two other studies to be conducted.

1. A muscle biopsy study which will assess the impact of resistance training on the skeletal muscle of people with first episode psychosis taking antipsychotics.
2. A qualitative study which will explore the experiences, barriers, and facilitators of participating in the resistance training intervention.

## Methods

### Study design

**Figure 1.**
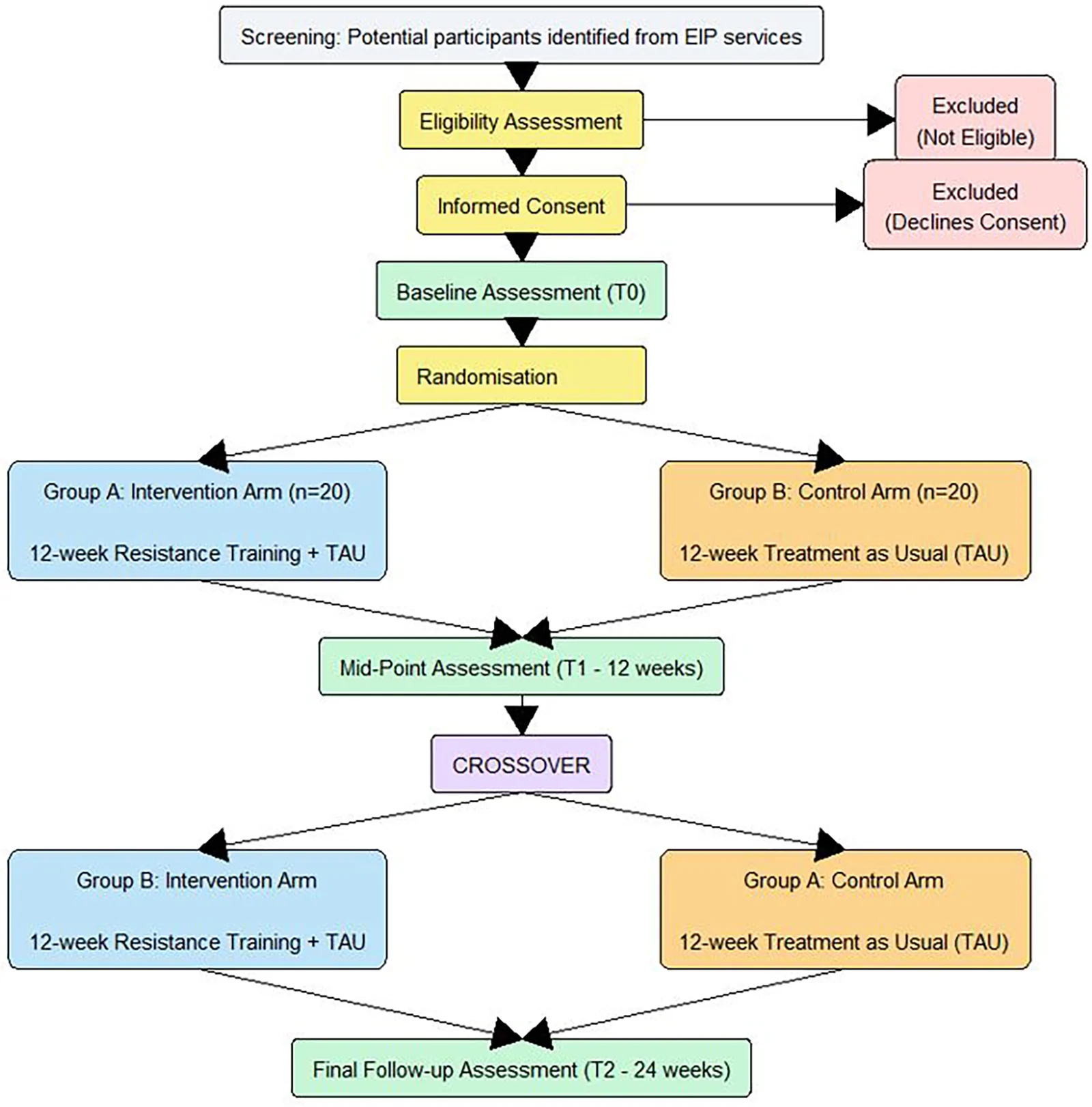
Flowchart of study timeline.

The study will be a multicentre, prospective, two-arm, crossover, randomised controlled feasibility trial. The study flow is outlined in Figure 1. Participants will be randomised 1:1 to either the Intervention Group (RT + TAU) or Control Group (TAU). At 12 weeks (T1), the control group will cross over to receive the 12-week intervention, while the initial intervention group returns to TAU (with no restrictions on exercise). The total study duration per participant is 24 weeks.

Assessments will take place at T0 (upon recruitment), 12 weeks (following completion of the resistance training programme and/or Treatment as Usual) and 24 weeks.

### Study setting

Recruitment will take place across two locations in Ireland: South Dublin and Cork City. Both locations have inpatient psychiatry wards and outpatient Early Intervention in Psychosis services. Participants who meet the above criteria and who are treated at the locations listed below will be marked as eligible for the study.

Inpatients: Patients who are admitted to the psychiatric inpatient units at:

• St Vincent’s University Hospital Elm Mount Unit,
• Cork University Hospital Adult Mental Health Unit,
• Mercy University Hospital St Michael’s Unit

Outpatients:

• RISE First episode psychosis service, Blackrock Hall PCC, Skehard Rd, Blackrock, Cork
• EIST First episode psychosis service, St Mary’s PCC, Gurranebraher, Cork
• Churchtown Community Mental Health Services, Unit 12, Nutgrove Retail Park, Dublin 14
• Milltown Mental Health Services, Milltown Road, Dublin 6
• Vergemount Outpatient building, Clonskeagh Hospital Campus, Clonskeagh, Dublin 6

### Inclusion criteria

Age 18–65 years.

Diagnosis of FEP (affective or non-affective) within the past 3 months.

Cumulative lifetime antipsychotic exposure <4 weeks.

Currently prescribed an antipsychotic.

Capacity to provide informed consent.

### Exclusion criteria

Medical conditions preventing safe exercise participation (e.g., unstable cardiovascular disease).

Pregnancy, as hormonal changes would increase the risk of musculoskeletal injury (due to relaxin increasing laxity in ligaments and joints), maternal and foetal cardiovascular strain during the Valsalva manoeuvre, and additional confounders (due to pregnancy’s effects on metabolism, energy levels, and physical capacity). Pregnancy is also associated with changes in body composition which affect the measurement of the primary outcome.

Concurrent participation in another clinical trial of an investigational medicinal product or device.

### Discontinuation criteria

Every effort will be made to continue the resistance training intervention, i.e. by switching to resistance bands in the event of an inpatient admission. If a participant’s condition changes so as to meet the exclusion criteria defined above, their participation in the intervention will be ceased until such time as they no longer meet the exclusion criteria.

Participant’s physical and mental health will be regularly monitored by a medical practitioner to ensure they remain eligible to receive the intervention.

### Informed consent

If permission is granted, a researcher will contact the individual to provide a full explanation of the study. This will take place in the medium most suitable to the participant, e.g. phone call, online meeting, or in-person. They will be given a Participant Information Leaflet and will have the chance to ask questions. Participants will also receive short videos explaining the study. The researcher will explain the potential benefits and negative effects of the intervention.

The consent process will emphasise that participation is entirely voluntary and that the participant can withdraw from the study at any time without any impact on their clinical care.

The researcher will ensure the participant understands:

• The purpose of the study.
• The procedures involved, including randomisation.
• The potential risks and benefits of participation.
• The commitment required.
• The procedures for data confidentiality and storage.

Separate consent will be sought for the main trial, the muscle biopsy sub-study, and the qualitative interview sub-study. A participant may consent to the main trial without consenting to the sub-studies. All participants will provide written informed consent before any study-specific procedures are performed.

### Screening, enrolment, and withdrawal

**Screening and Enrolment Log:** A comprehensive screening log will be maintained by all recruiting sites. This log will document every patient assessed for eligibility. For patients who are not subsequently randomised, the log will record the specific reason for non-enrolment (e.g., “Did not meet inclusion criterion #3”, “Met exclusion criterion #2”, “Declined to participate”, “Other”). This log will not contain patient-identifiable data.

**Procedure for Participant Withdrawal:** Participants will be free to withdraw from the trial at any time without giving a reason and without their medical care being affected. The investigator will make a reasonable effort to understand the reason for withdrawal for the trial record.

A distinction will be made between withdrawal from the **intervention** and withdrawal from the **trial (all follow-up)**.

• If a participant withdraws from the intervention, they will be asked for permission to continue collecting follow-up data and to attend the 12-week and 24-week assessment visits.
• If a participant wishes to withdraw from all follow-up, no further data will be collected, but data collected up to the point of withdrawal will be retained for the final analysis as per the initial consent. This will be clearly explained in the Participant Information Leaflet.

**Definition of Lost to Follow-up:** A participant will be considered lost to follow-up if they cannot be contacted after a minimum of three documented attempts by telephone and at least one attempt via email or post over a 4-week period.

### Interventions

Intervention Group (Weeks 1–12):

Participants will receive a 12-week, individualised, supervised RT programme consisting of two 60-minute sessions per week. Sessions include a warm-up, 45–50 minutes of resistance exercises (6–8 core exercises e.g., leg press, chest press), and a cool-down. Initial sessions will use resistance bands to introduce participants to exercise form and to allow participation in a variety of settings (inpatient and outpatient). Participants will then progress to free weights/machine weights in an appropriately equipped gym. The principle of progressive overload will be applied by increasing the weight when a participant can comfortably complete 3 sets of 10 repetitions on two consecutive sessions, although this principle will be adapted by the exercise supervisor to each individual’s needs. Participants will be allowed to bring a friend or family member to sessions for support, and will be given text and phonecall support to encourage attendance at the sessions. Attendance at the session and exercises performed will be logged by the instructor.

Control Group (Weeks 1–12):

Participants will receive Treatment as Usual (TAU) in their first-episode psychosis clinical teams. They will receive standard healthy lifestyle information but no structured exercise counselling. They crossover to receive the intervention at Week 13. Participants in the control group will not be encouraged to refrain from exercising.

The aerobic exercise of groups will be monitored through Fitbit activity and asking the participant to quantify any additional exercise performed.

After 24 weeks, both groups will be offered the opportunity to receive subsidised gym membership through HSE funding.

The timeline of the study for participants is displayed in Figure 1, and the timeline of assessments is displayed in Table 1.

**Table 1.** Schedule of assessments.

Assessment	Baseline (T0)	12 Weeks (T1)	24 Weeks (T2)
**Enrolment**			
Eligibility Screen	X		
Informed Consent	X		
Randomisation	X		
**Intervention**			
Resistance Training (Intervention Group)		<--- X --->	
Resistance Training (Control Group)			<--- X --->
**Assessments**			
Body Composition (Primary Outcome)	X	X	X
Height, Weight, BMI, Waist Circ.	X	X	X
Muscular Power	X	X	
Blood Pressure, Heart Rate	X	X	X
BPRS, GAF, EQ-5D, QPR	X	X	X
Food Diaries	X	X	X
Blood Sample (Metabolic/Endocrine)	X	X	X
Muscle Biopsy (Sub-study)	X	X	
Qualitative Interview (Sub-study)			X

Note: Fitbit data (activity/sleep) is collected continuously throughout the study period.

### Outcomes

Primary Outcome:

• **Feasibility:** Recruitment rates, retention (target ≥70% completing the programme), and adherence (target ≥65% of sessions). Attendance at a session will be marked if a participant completes over 50% of the scheduled exercises.
• **Acceptability:** We will use a 14-item self-report questionnaire similar to that used by Korman et al. ^8^ Responses to each item will be aggregated by summing item responses with reverse scoring for negative statements. Each item will be considered acceptable if ⩾65% of participants endorse the item.
• Change in **body fat percentage** from Baseline (T0) to 12 weeks (T1), assessed via Tanita MC-780MA body composition analyser by a blinded research-assistant.

Secondary Outcomes:

• **Physical Health:** Muscular power (3-rep max): Recorded by instructor
BMI: Tanita mc-780MA body composition analyser
Waist circumference: Recorded by research-assistant with measuring tape Resting heart rate: Measured by Fitbit
• **Mental Health & Functioning:** BPRS, GAF, EQ-5D, QPR scores: Scales performed by healthcare professional in the participant’s treating team.
• **Biomarkers:** Fasting serum lipids, glucose, HbA1c, CRP, IL-6, prolactin, testosterone, sex hormone-binding globulin.
• **Activity & sleep:** Using Fitbit watch.

Sub-studies:

1. **Biochemical Mechanisms:** Skeletal muscle biopsies (Vastus Lateralis) at T0 and T1 in a subsample (n = 24) to analyse protein expression (e.g., Akt/mTOR pathway, GLUT4).
2. **Qualitative Evaluation:** Semi-structured interviews with participants and instructors to explore barriers and facilitators.

### Sample size

As a feasibility study, the target sample size is **40 participants** (20 per arm). This is not powered for significance but chosen to estimate parameters (SD of outcome, recruitment rates) for a future larger RCT.

### Randomisation and blinding

Participants will be randomised (1:1) using a centralised web-based service. While the researcher will enrol participants, the centralised system will perform the assignment to prevent bias. Participants will be stratified by their prescribed antipsychotic’s propensity to lead to weight gain:

**Stratum 1:** Olanzapine, risperidone, quetiapine, paliperidone, amisulpride.

**Stratum 2:** Brexpiprazole, asenapine, aripiprazole, haloperidol, lurasidone, cariprazine.

Due to the nature of the intervention, participants and instructors cannot be blinded. However, outcome assessors for non-serum/biopsy markers will be blinded to group allocation. At the beginning of each follow-up assessment, participants will be reminded not to reveal their group allocation to the assessor. Any instances of unblinding will be recorded and reported.

### Statistical methods

• **Primary Analysis:** The primary analysis will use an Intention-to-Treat ANCOVA to compare change in body fat percentage at 12 weeks between groups, adjusting for baseline body fat percentage and medication stratum. Results will be reported as effect sizes (Cohen’s d) with 95% CIs. Statistical significance will be set at p < 0.05 and tests will be two sided.
• **Feasibility:** Descriptive statistics (rates with 95% CIs) for recruitment, retention, and adherence.
• **Sensitivity analyses:** A first sensitivity analysis will use a per-protocol analysis. A second sensitivity analysis will use Multiple imputation for the (ITT) population under a missing-at-random assumption.
• **Multiple Comparisons:** Given the exploratory nature of this feasibility study, p-values for secondary outcomes will be interpreted with caution.

### Data management and monitoring

Data will be collected on paper CRFs and entered into a secure, password-protected electronic database. Anonymised data will be stored on encrypted university servers in compliance with GDPR. The study will adhere to FAIR data principles. Due to the low-risk nature of the RT intervention and small sample size, a formal independent Data Monitoring Committee was deemed unnecessary by the Trial Steering Committee. To ensure the trial is running adequately biannual audits of the site master files and consent forms will be performed.

## Patient and public involvement

PPI experts were consulted prior to writing this protocol, and will continue to be involved in the delivery, writing, and dissemination of the study.

## Ethics and dissemination

Ethical approval will be obtained from the Clinical Research Ethics Committee of the Cork Teaching Hospitals and the UCD Research Ethics Committee. Written informed consent will be obtained for the main trial and separately for sub-studies. Results will be disseminated via peer-reviewed publications, conferences, and lay summaries co-produced with PPI partners. Any substantial modifications to the protocol will be submitted for approval to the relevant ethics committees and updated on the trial registry.

## Data Availability

No underlying data are associated with this article. This is a protocol for a randomised controlled feasibility trial and no datasets were generated or analysed for this manuscript. The full de-identified participant-level datasets generated during the trial, including variables (age, sex, BMI, and clinical scores), values behind reported means and standard deviations, and the data points used to generate all figures, will be made available upon the trial’s completion, upon reasonable request. In accordance with the FAIR principles and GDPR compliance, these data will be deposited in the UCD **Research Data Repository.**
•
**Repository Name:** UCD Research Data •
**Persistent Identifier**: https://doi.org/10.5281/zenodo.18214167 ^10^
•
**Licence:** Creative Commons Attribution 4.0 International (CC-BY 4.0) •
**Repository Name:** UCD Research Data •
**Persistent Identifier**: https://doi.org/10.5281/zenodo.18214167 ^10^
•
**Licence:** CC-BY 4.0 This repository contains the following reporting materials:
• Full SPIRIT 2025 Checklist • Sample Participant Information Leaflet and Informed Consent Form • Detailed Statistical Analysis Plan (SAP)
