# Supplementary material for: Strategic Timing of Resistance Training to Guard Against Antipsychotic-Induced Metabolic Syndrome (START GAAIMS): A Feasibility Study Protocol
Source: HRB Open Res. 2026 Apr 30;9:47. [Version 1] doi: 10.12688/hrbopenres.14320.1 (PMC13560837; doi:10.12688/hrbopenres.14320.1)
Supplement: Peer review history

# Reviewer report: Reviewer response for version 1

**DOI:** 10.21956/hrbopenres.15763.r56967

**Published:** 2026-08-18

**Authors:** Scott Teasdale

**Affiliations:** 1 University of New South Wales, Sydney, New South Wales, Australia

This protocol addresses an important and clinically relevant question and the focus on drug-naïve or minimally treated participants is a particular strength. The intervention is clearly described, and the inclusion of physical, metabolic, psychological and feasibility outcomes provides a valuable foundation for a larger trial.

 Abstract and Introduction:

 1. As described, this appears to be a randomised wait-list-controlled feasibility trial rather than a traditional cross-over trial. Please justify the “cross-over” designation and explain how it is reflected in the outcome collection and analysis. In particular, clarify what is measured in the original intervention group at 24 weeks and how these data contribute to the cross-over comparison.

 2. Consider describing the study as assessing the feasibility of the intervention model rather than the feasibility of the trial design.

 Methods:

 3. Clarify how first-episode psychosis was confirmed, e.g., psychiatrist-determined.

 4. Clarify definition of antipsychotic naïve as criteria includes people needing to be prescribed antipsychotic medication. 

 5. Please state the medical-clearance to exercise process.

 6. Explain how participants’ capacity to provide informed consent will be assessed and confirmed, e.g., determined by treating team or UBACC

 7. The statement that participants’ physical and mental health will be “regularly monitored” is vague. Please specify further, e.g., timing, who's responsible, criteria for pausing or discontinuing participation.

 8. State where the resistance-training sessions will occur and describe the qualifications, training and level of supervision provided by the exercise professional.

 9. Describe any measures used to support attendance and retention, such as reminders, travel assistance or other practical supports.

 10. Provide further detail on outcome measure procedures, e.g., assessor training, assessment timepoints, standardisation procedures and appropriate supporting references.

 11. If possible, expand the description of the semi-structured interviews, e.g., the underlying theory, frameworks informing the interviews, who will conduct them, when they will occur, the topics covered, recording and transcription procedures, and the proposed analytical approach.

 12. Justify the proposed sample size with reference to relevant guidance or comparable feasibility studies.

 13. Provide further detail on patient and public involvement, including who was involved, the extent and timing of involvement, and how this feedback shaped the intervention and trial procedures.

 14. Clarify how adverse events and other potential harms will be identified, recorded, assessed, managed and reported, including any stopping or escalation procedures.

Is the study design appropriate for the research question?

Yes

Is the rationale for, and objectives of, the study clearly described?

Yes

Are sufficient details of the methods provided to allow replication by others?

No

Are the datasets clearly presented in a useable and accessible format?

Not applicable

Reviewer Expertise:

Mental health services, lifestyle interventions

I confirm that I have read this submission and believe that I have an appropriate level of expertise to confirm that it is of an acceptable scientific standard, however I have significant reservations, as outlined above.

# Reviewer report: Reviewer response for version 1

**DOI:** 10.21956/hrbopenres.15763.r56961

**Published:** 2026-08-17

**Authors:** Nicole Laurent

**Affiliations:** 1 Independent Researcher, Vancouver, Washington, USA

O’Mahony and colleagues propose a multicenter, two arm, crossover randomized feasibility trial of a twelve-week supervised resistance training program in forty adults with first episode psychosis and less than four weeks of cumulative lifetime antipsychotic exposure, recruited from inpatient units and outpatient mental health services in South Dublin and Cork City. The rationale is sound, and the timing question is clinically important. Prior interventions delivered during minimal antipsychotic exposure have combined exercise with broader lifestyle support, while the exercise focused studies in first episode psychosis have generally used aerobic or mixed modality programs. Testing supervised resistance training as a discrete intervention during the earliest phase of antipsychotic treatment attempts to address this gap. That is valuable.  

 The design carries several strengths. Stratifying randomization by antipsychotic weight gain propensity reduces the risk that agents with greater metabolic liability will be unevenly distributed between groups. Allowing resistance bands during inpatient admission preserves intervention continuity when gym access is disrupted. The delayed intervention and subsidized gym membership balance participant burden with access to potential benefit. The muscle biopsy study adds mechanistic depth by examining skeletal muscle signaling alongside body composition and muscular performance. My central reservation is that the protocol can determine whether its recruitment, retention, adherence, and acceptability thresholds are met, but is less able to explain why. The protocol records reasons for nonenrolment and withdrawal and collects participant feedback at week 24, but these measures will not distinguish program limitations from symptoms and medication effects that shape participation during early antipsychotic treatment.

 1. Antipsychotic adverse effects that directly obstruct exercise participation are not measured. Safety and acceptability are stated objectives, yet no standardized adverse event collection framework is specified for either. The protocol includes no assessment of sedation, akathisia, antipsychotic induced parkinsonism, or orthostatic symptoms. Regular monitoring by a medical practitioner is described in terms of continued eligibility, which does not generate structured data capable of entering the feasibility analysis. The SPIRIT checklist nevertheless reports that harms are defined and assessed on protocol page 6 and that statistical methods for harms appear on page 9, although neither the protocol nor the statistical analysis plan provides either framework. Participants enter within four weeks of antipsychotic initiation, when sedative adverse events cluster and acute akathisia commonly emerges, and both can limit attendance and the controlled movement required for resistance training. Blood pressure is recorded without an orthostatic protocol. Stratification is based on weight gain propensity and does not track akathisia burden, with olanzapine and quetiapine in Stratum 1 and the partial agonists in Stratum 2. A missed or partial session is therefore recorded without a structured adverse effect measure capable of explaining it. Adding the Barnes Akathisia Rating Scale and the Simpson-Angus Scale, or other validated instruments of the authors’ choice, alongside brief measures of sedation and orthostatic symptoms would allow adherence and safety findings to be interpreted against the treatment effects most likely to limit participation.

 2. Antipsychotic dose is not recorded. The medication stratum is assigned at randomization and enters the primary ANCOVA as a covariate. The protocol does not state that dose is recorded, nor does it explain how treatment changes will be documented. Weight gain and prolactin elevation vary with dose to differing degrees across agents, so a stratum fixed at baseline cannot represent exposure across twelve weeks. A participant switched from olanzapine to aripiprazole at week five will remain classified in Stratum 1, even when the switch follows emerging weight gain or another adverse effect relevant to the study outcomes. The authors should record the antipsychotic and dose at each assessment. Any switch or change in formulation should also be dated. This would allow adherence and body fat change to be interpreted against actual pharmacological exposure. An equivalent dose could be derived later if needed. The first reviewer asked that switching be recorded, and dose belongs with it.

 3. The proposed GLUT4 mechanism is not matched by a systemic measure of insulin resistance. The introduction identifies improved glucose regulation through skeletal muscle GLUT4 as part of the rationale for resistance training, and the biopsy sub-study measures GLUT4 protein expression. The blood panel includes fasting glucose and HbA1c but no fasting insulin, leaving the protocol without a systemic measure of insulin resistance against which the muscle finding can be interpreted. In antipsychotic naive first episode schizophrenia, fasting insulin and HOMA IR are elevated even when HbA1c is unchanged. Adding fasting insulin to the scheduled blood panel would permit calculation of HOMA IR from the glucose already being collected and align the circulating metabolic assessment with the mechanism the authors have made central to the trial.

 4. The nutritional conditions required by the proposed mechanism are not specified or evaluated. The introduction and abstract do more than present resistance training as a pragmatic intervention for antipsychotic associated weight gain. They argue that resistance training can direct the increased caloric intake produced by antipsychotic treatment toward muscle protein synthesis rather than adipose storage. That mechanism depends on the nutritional substrate available during training, particularly total energy and protein intake, yet the protocol specifies no protein target, dietetic support, or structured nutritional component. Food diaries are scheduled at baseline, week 12, and week 24, but their duration, method of analysis, and place within the outcomes are not stated. The trial can estimate the effect of resistance training under usual dietary conditions, but it cannot test the nutritional mechanism it presents or determine whether differences in body composition response track differences in energy and protein intake. The authors should state how the diaries will be collected and analyzed for these variables and how the findings will be integrated with the body composition results. If the dietary data are not intended to evaluate this premise, the claim that resistance training redirects antipsychotic driven caloric surplus toward muscle protein synthesis should be moderated.

 5. The participant information and consent materials do not match the protocol. The participant leaflet understates what participation involves and conflicts with the protocol in several substantive areas. It presents the mental health assessments as taking no more than two minutes, although the BPRS is a clinician-rated interview scheduled alongside the GAF, EQ-5D, and QPR at each appointment. It describes recent food intake as a further two-minute assessment, which does not correspond to the food diaries in the schedule of assessments, and the protocol states no diary duration. Explanation of the blood samples are only in terms of sugar and fat metabolism without disclosing the fasting requirement or the inflammatory and endocrine analytes being measured. The description of the assessment visits omits waist circumference, blood pressure, heart rate, and the three repetition maximum test. The protocol also states that the distinction between withdrawing from the intervention and withdrawing from all follow up, including retention of previously collected data, will be clearly explained in the leaflet, but that explanation is absent. The biopsy materials conflict over the anatomical site and over whether stored tissue is restricted to the present study or may be used for unrelated research, including projects conducted by commercial or pharmaceutical researchers. The named ethics committees and approval status are not reconciled, while the leaflet retains an incomplete video link and unresolved template instructions. These materials should be revised so that participants receive an accurate account of the procedures, burden, withdrawal options, and permitted uses of their tissue before consent is obtained.

 6. Muscular power is named as a secondary outcome and measured as maximal strength. The protocol lists muscular power among the physical health secondary outcomes and specifies a three repetition maximum as the measure. A 3RM assesses maximal dynamic strength to be evaluated in relation to movement velocity, and no measure capturing velocity appears anywhere in the protocol. The construct should be renamed as muscular strength or the measure should change. The 3RM is recorded by an instructor, and the protocol states that instructors cannot be blinded to allocation while assessors for all other nonserum and nonbiopsy outcomes will be. Table 1 schedules this measure at baseline and week 12 alone, so participants crossing over at week 13 complete the intervention with no postintervention assessment of it.

 7. Bioelectrical impedance is used for the primary body composition outcome without stated standardization procedures. Change in body fat percentage is measured with the Tanita MC-780MA. Impedance estimates vary with hydration, yet the protocol gives no preparation conditions for this assessment. It does not specify the time of day or provide hydration instructions, and no interval is required between the most recent training session and measurement. The fasting requirement applies to blood collection but is not extended to the body composition assessment. The participant leaflet describes the measurement as taking less than one minute and provides no preparation instructions. Adjustment for baseline body composition in the statistical analysis plan addresses differences between participants, not variation in measurement conditions within the same participant. The authors should specify standardized assessment conditions because the preliminary effect size estimate this trial exists to produce will be derived from this measure and used to plan the definitive trial.

 9. The qualitative sub-study will not capture participants who withdraw from all follow up. Table 1 schedules semi structured interviews with participants and instructors at week 24 only. Figure 1 contains no qualitative contact before the final follow-up. Participants who stop the intervention may agree to remain in follow up, but those who withdraw from all follow up cannot contribute an interview. The protocol asks the investigator to make a reasonable effort to record the reason for withdrawal, which provides the research team’s documentation rather than the participant’s own account of what prevented continuation. The sub-study is intended to explain the barriers and facilitators underlying the feasibility results, yet the participants least able to remain in the trial will not be heard from. The authors should state how the experiences of participants who discontinue all follow up will be captured.

 START GAAIMS asks a clinically important question at the point when prevention is most likely to matter. The remaining concerns do not require a larger or more elaborate study. They require the protocol to separate failure of the intervention from factors arising outside the intervention itself, and to reconcile the specific places where the protocol and supporting materials define the trial differently or understate what participation involves. Feasibility estimates are useful only when noncompletion can be interpreted, while a preliminary effect size is useful only when its measurement is dependable. These are correctable problems. Addressing them would give the definitive trial a credible empirical foundation rather than a set of parameters whose meaning remains uncertain.

Is the study design appropriate for the research question?

Partly

Is the rationale for, and objectives of, the study clearly described?

Yes

Are sufficient details of the methods provided to allow replication by others?

Yes

Are the datasets clearly presented in a useable and accessible format?

Not applicable

Reviewer Expertise:

clinical psychology, metabolic psychiatry, nutrition, clinical intervention, qualitative research, psychiatric recovery

I confirm that I have read this submission and believe that I have an appropriate level of expertise to confirm that it is of an acceptable scientific standard, however I have significant reservations, as outlined above.

## References

[1] : Sex hormone levels in drug-naïve, first-episode patients with psychosis. *International Journal of Psychiatry in Clinical Practice* .2020;24(1) : 10.1080/13651501.2019.169911720-24 10.1080/13651501.2019.1699117 31825684

[2] : Prolactin and morning cortisol concentrations in antipsychotic naïve first episode psychosis: A systematic review and meta-analysis. *Psychoneuroendocrinology* .2023;150: 10.1016/j.psyneuen.2023.106049 10.1016/j.psyneuen.2023.106049 36758330

[3] : Impaired Glucose Homeostasis in First-Episode Schizophrenia. *JAMA Psychiatry* .2017;74(3) : 10.1001/jamapsychiatry.2016.3803 10.1001/jamapsychiatry.2016.3803 PMC635295728097367

[4] : Antipsychotic-Related Prolactin Changes: A Systematic Review and Dose–Response Meta-analysis. *CNS Drugs* .2025;39(10) : 10.1007/s40263-025-01218-z937-947 10.1007/s40263-025-01218-z PMC1242323740830715

[5] : Antipsychotic-Induced Weight Gain: Dose-Response Meta-Analysis of Randomized Controlled Trials. *Schizophrenia Bulletin* .2022;48(3) : 10.1093/schbul/sbac001643-654 10.1093/schbul/sbac001 PMC907742635137229

[6] : A RATING SCALE FOR EXTRAPYRAMIDAL SIDE EFFECTS. *Acta Psychiatrica Scandinavica* .1970;45(S212) : 10.1111/j.1600-0447.1970.tb02066.x11-19 10.1111/j.1600-0447.1970.tb02066.x 4917967

[7] : Antipsychotic Pharmacotherapy and Orthostatic Hypotension. *CNS Drugs* .2011;25(8) : 10.2165/11591710-000000000-00000659-671 10.2165/11591710-000000000-00000 21790209

[8] : A Rating Scale for Drug-Induced Akathisia. *British Journal of Psychiatry* .1989;154(5) : 10.1192/bjp.154.5.672672-676 10.1192/bjp.154.5.672 2574607

[9] : The Assessment and Treatment of Antipsychotic-Induced Akathisia. *The Canadian Journal of Psychiatry* .2018;63(11) : 10.1177/0706743718760288719-729 10.1177/0706743718760288 PMC629918929685069

[10] : The trajectory of sedative adverse events caused by antipsychotics: a meta-analysis of individual participant data from randomised, placebo-controlled, clinical trials in acute phase schizophrenia. *The Lancet Psychiatry* .2025;12(4) : 10.1016/S2215-0366(25)00025-2266-275 10.1016/S2215-0366(25)00025-2 40113354

[11] : The feasibility of resistance training versus aerobic exercise in a rehabilitation setting for people living with psychotic disorders: A randomised controlled trial. *Australian & New Zealand Journal of Psychiatry* .2026;60(6) : 10.1177/00048674251361681538-552 10.1177/00048674251361681 41215676 PMC13191080

[12] : Exercise training complementary to specialised early intervention in patients with first-episode psychosis: a feasibility randomised trial. *Pilot and Feasibility Studies* .2021;7(1) : 10.1186/s40814-021-00900-5 10.1186/s40814-021-00900-5 34412705 PMC8375206

[13] : Exercise as an intervention for first‐episode psychosis: a feasibility study. *Early Intervention in Psychiatry* .2018;12(3) : 10.1111/eip.12329307-315 10.1111/eip.12329 PMC600179626987871

[14] : Evaluating an individualized lifestyle and life skills intervention to prevent antipsychotic‐induced weight gain in first‐episode psychosis. *Early Intervention in Psychiatry* .2016;10(3) : 10.1111/eip.12230267-276 10.1111/eip.12230 25721464

[15] : Interventions to prevent antipsychotic-induced weight gain and metabolic complications in individuals with a first-episode psychosis and minimal antipsychotic exposure: a systematic review and meta-analysis. *Psychological Medicine* .2026;56: 10.1017/S0033291726104139 10.1017/S0033291726104139 PMC1307922341958303

# Reviewer report: Reviewer response for version 1

**DOI:** 10.21956/hrbopenres.15763.r55212

**Published:** 2026-06-02

**Authors:** Dongyu Kang

**Affiliations:** 1 Hartford Hospital, Hartford, USA

O’Mahony et al. presented a study protocol for a multicenter, two-arm, crossover, randomized controlled feasibility trial. Forty adults (18–65 years) with FEP and < 4 weeks of antipsychotic exposure will be recruited from services in Dublin and Cork. Participants will be randomised (1:1) to an Intervention Group (12-week supervised RT programme + Treatment as Usual [TAU]) or a Control Group (TAU only). After 12 weeks, the control group will cross over to receive the intervention. The primary outcome is the feasibility of the trial design (recruitment, retention, adherence) and a preliminary estimate of the effect size for change in body fat percentage at 12 weeks. Secondary outcomes include muscular power, metabolic biomarkers (glucose, lipids, inflammatory markers), and psychiatric symptoms (BPRS). This study will determine preliminary measures of the effects of

 resistance training on body composition in individuals with FEP starting antipsychotic medication, as well as its feasibility and acceptability in this cohort. Findings will inform the design of a definitive RCT to establish RT as a standard co-treatment for metabolic health in early psychosis. This topic is of substantial clinical importance. This protocol is generally well written with a solid study design. However, I have several comments for the authors to consider.

  

1. In the abstract and introduction, a brief description of resistance training (for example, any exercise where muscles contract against an external force to build strength, muscle mass, and endurance) would be helpful for future readers who might not be familiar with the study background.
2. In the protocol, authors used ‘body fat percentage’, ‘body composition’, and ‘lean body mass’ referring to different but similar or overlapping concept. A sentence defining these concepts and their relationship in the introduction would benefit the readers.
3. In the abstract, the ‘RCT’ and ‘RT’ need to be defined before using the acronym.
4. In the Introduction, a strengthened logic link between the metabolic dysregulation to the resistance training would improve the protocol. For example, in the first paragraph of the introduction, authors ended with ‘appetite and impair glucose metabolism’, however the current study does not focus on this topic, but body composition. Therefore, a link between the ‘problem’ and ‘solution’ is missed. Alternatively, authors could emphasize more on the body weight and body composition changes induced by antipsychotics and diagnose of psychotic disorders in the introduction.
5. In the Introduction, one missing element is the current treatment or intervention for metabolic dysregulation in psychotic disorder. What gap will the current study fill in this topic? Such factor would strengthen the urgent needs for a novel treatment, such as resistance training.
6. For the inclusion criteria, could the authors elaborate on their thought about the age range in this study. Specifically, why authors set the upper limit to 65 years old? One might concern about the effect of age on glucose and lipid metabolism. Also, age might influence the effect and safety of resistance training. Such concern suggested age might be a confounding factor to this research.
7. For the exclusion criteria, it would be helpful to clarify whether participants comorbid with other conditions will be recruited as well? Such conditions might confound the effect of resistance training. For example, individuals comorbid with type I diabetes might respond differently to those who without. Also, individuals comorbid with intellectual disability would face different and specific challenges during the study.
8. For the outcomes, I would recommend to record appetite and food diary in this study, if applicable. The metabolic side effect of antipsychotics is associated with a rapid increase of appetite and food intake. It would help for future data interpretation if record appetite and food diary. Following this idea, I would recommend authors to include cigarette, alcohol, and illicit drug consumption as well.
9. In the Secondary Outcomes, full name of the scale is needed before using acronym. Also, adding reference to each scale used would be preferred.
10. In the Randomization and blinding, Stratum randomization, will the study only recruit those who are taking enlisted medication? If so, please add this to inclusion/exclusion criteria.
11. What Stratum would be used if the participant is receiving multiple antipsychotics? This might not happen as for the FEP usually receive single antipsychotic in the beginning of their treatment, but it clarifying this would improve the rigor in the protocol.
12. Would authors collect the medical record associated with the study? For example, if participant switch antipsychotic during the study period, it would be helpful if such information is recorded.

Is the study design appropriate for the research question?

Yes

Is the rationale for, and objectives of, the study clearly described?

Yes

Are sufficient details of the methods provided to allow replication by others?

Partly

Are the datasets clearly presented in a useable and accessible format?

Not applicable

Reviewer Expertise:

My areas of research are metabolic side effect of antipsychotics and developing novel neural circle based intervention in psychotics using neuromodulation and neuroimaging methods.

I confirm that I have read this submission and believe that I have an appropriate level of expertise to confirm that it is of an acceptable scientific standard.
